# Comprehensive studies on the stability of yogurt-type fermented soy beverages during refrigerated storage using dairy starter cultures

**DOI:** 10.3389/fmicb.2023.1230025

**Published:** 2023-08-25

**Authors:** Małgorzata Ziarno, Dorota Zaręba, Iwona Ścibisz, Mariola Kozłowska

**Affiliations:** ^1^Department of Food Technology and Assessment, Institute of Food Science, Warsaw University of Life Sciences – SGGW (WULS-SGGW), Warsaw, Poland; ^2^Professor E. Pijanowski Catering School Complex in Warsaw, Warsaw, Poland; ^3^Department of Chemistry, Institute of Food Science, Warsaw University of Life Sciences – SGGW (WULS-SGGW), Warsaw, Poland

**Keywords:** starter cultures, plant-based beverages, acidification, microflora, texture, carbohydrates

## Abstract

**Introduction:**

This study aimed to assess the feasibility of utilizing commercially available dairy starter cultures to produce yogurt-type fermented soy beverages and evaluate the fundamental properties of the resulting products.

**Methods:**

Sixteen different starter cultures commonly used in the dairy industry for producing fermented milks, such as yogurt, were employed in the study. The study investigated the acidification curves, acidification kinetics, live cell population of starter microflora during refrigerated storage, pH changes, water-holding capacity, texture analysis, carbohydrates content, and fatty acid profile of the yogurt-type fermented soy beverage.

**Results and Discussion:**

The results demonstrated that the starter cultures exhibited distinct pH changes during the fermentation process, and these changes were statistically significant among the cultures. The acidification kinetics of different cultures of lactic acid bacteria showed characteristic patterns, which can be used to select the most suitable cultures for specific product production. The study also revealed that the choice of starter culture significantly influenced the starter microorganisms population in the yogurt-type fermented soy beverage. Additionally, the pH values and water-holding capacity of the beverages were affected by both the starter cultures and the duration of refrigerated storage. Texture analysis indicated that storage time had a significant impact on hardness and adhesiveness, with stabilization of these parameters observed after 7–21 days of storage. Furthermore, the fermentation process resulted in changes in the carbohydrate content of the soy beverages, which varied depending on the starter culture used.

## 1. Introduction

Humans have consumed fermented foods for thousands of years. Various fermented animal and vegetable products, spanning from yogurt and pickles to kumis and wine, miso, and fermented sausages can be found all over the globe from Africa to Europe, South America to Asia. The activity of bacteria and yeasts involved in lactic and alcoholic fermentation results in unique taste and flavors. The dairy industry has many years of experience in the production of fermented products and utilizing specific strains of lactic acid bacteria. The consumption of dairy yogurt has been associated with several health benefits, including improved gut health, enhanced nutrient absorption, and a boosted immune system. These advantages are primarily attributed to the presence of lactic acid bacteria such as *Lactobacillus delbrueckii* subsp. *bulgaricus* and *Streptococcus thermophilus*, commonly used as starter cultures in yogurt production (Settachaimongkon et al., 2014; Narvhus and Abrahamsen, 2022). Given the established role of these cultures in dairy fermentation, their application in creating yogurt-like soy beverages shows potential for delivering a similar product experience. This knowledge can be leveraged to enhance the quality and efficiency of producing plant-based yogurts, including soy-based options. Soy is one of the most widely cultivated crops globally (Shurtleff and Aoyagi, 2013). It serves as animal feed and is also consumed by humans in various forms, such as soy flour, tofu, meat and coffee substitutes, and soy beverages (Berk, 1992; Huang, 2008).

In today’s world, people are becoming increasingly mindful of the food they consume. Movements such as vegetarianism and veganism, driven by a broader concern for animal welfare, reject the consumption of meat. Additionally, regular consumers are constantly seeking out new and intriguing dishes. Consequently, it is worthwhile to explore alternative plant-based foods for everyday meals (McClements and Grossmann, 2021; Alae-Carew et al., 2022). These alternatives offer the advantage of requiring fewer resources for production while providing higher yields (Laassal and Kallas, 2016; Woodside et al., 2016). The growing demand for plant-based substitutes for dairy products has spurred the development of innovative food technologies capable of replicating the taste, texture, and nutritional composition of traditional dairy products. Manufacturers of plant-based dairy alternatives are primarily focused on improving the taste and texture of their products to replicate and replace fermented milks with plant-based substitutes. However, there are challenges that need to be overcome, including nondairy aftertastes, difficulties with plant protein bioavailability, as well as the content and bioavailability of minerals and vitamins (Mäkinen et al., 2015; Clegg et al., 2021; Leonard et al., 2022; Pua et al., 2022; Sugahara et al., 2022). These aspects should be considered as part of future research when exploring the use of dairy starter cultures for obtaining yogurt-type fermented soy beverages. Research conducted thus far indicates that the utilization of carefully selected dairy starter cultures has proven effective in addressing these challenges (Blagden and Gilliland, 2005). Among these alternatives, yogurt-type fermented soy beverages have gained considerable popularity due to their potential health benefits and suitability for individuals with lactose intolerance or dairy allergies (Madsen et al., 2021; Montemurro et al., 2021; Vieira et al., 2021). To achieve the desired sensory attributes and ensure consistent product quality, employing dairy starter cultures in the production of these soy-based beverages has emerged as a promising approach.

It is possible to utilize lactic acid bacteria during the production of yogurt-type fermented soy beverage to achieve the desired sensory characteristics. Moreover, incorporating these lactic acid bacteria strains can influence the chemical composition of yogurt-type fermented soy beverage, enhancing its nutritional value while reducing fermentation time (Ziarno et al., 2019). Introducing controls into the yogurt-type fermented soy beverage production process will also ensure a more stable and reproducible product. This is especially crucial in industrial production, where maintaining consistent product quality and predictable processes are essential. The selection of suitable starter cultures is crucial for achieving the desired sensory properties. It’s important to consider other factors, such as the availability of cultures specifically designed for plant-based beverages. Dairy starter cultures are widely available on the market and are widely used in industrial fermentation processes, including fermented milks. These starter cultures can be more cost-effective to use as they are readily available and may be less expensive compared to cultures specifically designed for plant-based beverages. Although dairy starter cultures are not specifically tailored for plant-based beverages, there is a possibility that they may adapt well to the fermentation conditions of soy beverages. Some of these starter cultures may be able to convert the carbohydrate components in soy beverages into suitable fermentation products, thus affecting the final quality and taste of the yogurt-type fermented soy beverage. This topic is of interest due to its potential for applying the experiences and technologies employed in the dairy industry to improve the quality of yogurt-type fermented soy beverage production. In recent years, there has been a growing interest in plant-based products, including yogurt-type fermented soy beverage, which serves as a popular alternative to yogurt. However, compared to yogurt, the production of yogurt-type fermented soy beverage is still considered a rudimentary process (Madsen et al., 2021; Montemurro et al., 2021; Vieira et al., 2021).

Therefore, the aim of this study was to determine the feasibility of utilizing commercially available dairy starter cultures for producing yogurt-type fermented soy beverages and evaluating the fundamental properties of the resulting products after fermentation and during subsequent storage. By incorporating lactic acid bacteria commonly used in the dairy industry, the aim is to elevate the production of yogurt-type fermented soy beverage from a rudimentary process to a fully controlled industrial process. We will focus on monitoring the changes that occurred during the storage period rather than analyzing the changes during the fermentation process. We will delve into the scientific aspects underlying this approach, highlighting the key factors that influence the fermentation process and contribute to the characteristics of the final product. By comprehending the advantages and challenges associated with employing dairy starter cultures, we can gain valuable insights into the potential for enhancing the sensory profile, texture, and nutritional value of yogurt-type fermented soy beverage.

## 2. Materials and methods

### 2.1. Materials

The study utilized 16 different starter cultures commonly employed in the dairy industry for the production of yoghurt. All of these cultures were in lyophilized form and purchased from local distributors. Among them, nine dairy starter cultures (YC-X16, YC-380, YO-820, Y107, YO-D, YO-S, YO-B, YO-122, and YC-180) consisted of typical yogurt microorganisms, including *L. delbrueckii* species and *S. thermophilus* species. Four dairy starter cultures (ABY-3, YOMIX-205, YOMIX-207, and LBA) contained not only *L. delbrueckii* and *S. thermophilus* but also additional microflora in the form of bifidobacteria and/or *Lactobacillus acidophilus*. The remaining three dairy starter cultures (XPL-1, LCP, and LCR) consisted of monocultures of lactobacilli or a mixture of different lactic acid bacteria, including *Lactobacillus* spp., *Leuconostoc* spp., *Lactococcus* spp., etc. The selected dairy starter cultures varied in their declared flavor properties, viscosity properties, and acidifying properties, which are listed in Table 1 along with the culture names, producer names, full species composition as declared by the producers, and the characteristics of the resulting fermented beverages. These starter cultures, commonly used in the dairy industry for yogurt and other fermented dairy product production, can impart the desired flavors, smells and rheological features. By using commercial dairy starter cultures, a traditional soy beverages that tastes reminiscent of classic fermented milks can be achieved.

**Table 1:** Dairy starter cultures used in the experiments and basic information declared by their manufacturers.

Ref.	Producer	Full name	Composition	Form	Flavor properties	Viscosity properties	Acidifying properties
YC-X16	Chr. Hansen	YC-X16 - Yo-Flex®	*Streptococcus thermophilus*, *Lactobacillus delbrueckii* subsp. *bulgaricus*	Freeze-dried culture	Mild	High	Weak
YC-380		YC-380 - Yo-Flex®			Intensive	Medium	Medium
YO-820	CSK Food Enrichment	Ceska®-star Y 820			Intensive	Medium	High
Y107		Ceska® Y 107			Medium	High	Medium
YO-D	Mediterranea Biotecnologie	YO-D			Very mild	Very low	Medium
YO-B		YO-B			Very mild	Low	High
YO-S		YO-S			Mild	Low	Medium
YO-122	Danisco	Y 122			Mild	Low	Medium
YC-180	Chr. Hansen	YC-180 Yo-Flex®	*Streptococcus thermophilus*, *Lactobacillus delbrueckii* subsp. *bulgaricus*, *Lactobacillus delbrueckii* subsp. *lactis*		Medium	High	Medium
ABY-3		ABY-3 Probio-Tec®	*Streptococcus thermophilus*, *Lactobacillus delbrueckii* subsp. *bulgaricus*, *Lactobacillus acidophilus*, *Bifidobacterium species*	Freeze-dried culture	Very mild	High	Very weak
YOMIX-205	Danisco	YO-MIX™ 205	*Streptococcus thermophilus, Lactobacillus delbrueckii* subsp. *bulgaricus*, *Lactobacillus acidophilus*, *Bifidobacterium lactis*		Mild	High	Weak
YOMIX-207		YO-MIX™ 207			Mild	High	Weak
LBA	Mediterranea Biotecnologie	LBA	*Streptococcus thermophilus*, *Lactobacillus bulgaricus*, *Lactobacillus acidophilus*, *Bifidobacterium lactis*		Medium	High	Medium
XPL-1	Chr. Hansen	XPL-1 – eXact® Plus	*Lactococcus lactis* subsp. *lactis*, *Lactococcus lactis* subsp. *cremoris*, *Leuconostoc species*, *Lactococcus lactis* subsp. *lactis* biovar *diacetylactis*, *Streptococcus thermophilus*	Freeze-dried culture	Intensive	High	Medium
LCP	Mediterranea Biotecnologie	LCP	*Lacticaseibacillus casei* subsp. *paracasei*		Mild	Low	Very weak
LCR		LCR	*Lacticaseibacillus casei* subsp. *rhamnosus*		Mild	Low	Very weak

The raw soy beverage was obtained by a local soy beverage company by hot mashing soybeans using appropriate technological methods and then mixing them with drinking water in a weight ratio of 1:2, and finally in a weight ratio of 1: 9. The soy beverage samples, used in the experiments, had a fat content of 1.57% ± 0.05, a protein content of 1.85% ± 0.13, a carbohydrate content of 1.47 ± 0.11, and a total solids content of 4.90% ± 0.74.

### 2.2. Fermentation of soy beverages

Before the experiments, the soy beverage underwent sterilization at 121°C for 15 min using an autoclave (Systec DE-45, Systec GmbH & Co. KG, Germany). The dairy starter cultures were stored according to the manufacturer’s instructions at −25°C. Before their use in the experiments, the dairy starter cultures were rehydrated by placing them in a small portion of soy beverage at 30°C for 20 min. They, they were added to an appropriate amount of soy beverage heated to 45°C, ensuring that their content was 0.06% by weight. The soy beverage samples, inoculated with the dairy starter cultures, were then transferred to sterile 170 mL glass jars equipped with metal caps.

To determine the acidification curves and acidification kinetics, these jars were placed in a laboratory incubator set at 37°C for 14 h, allowing the fermentation process to occur. Throughout this time, samples were taken at regular intervals of 60 min to determine the acidification curve.

In order to obtain samples for storage research, further samples were acquired, prepared, and subjected to the same fermentation process. However, in this case, the fermentation was conducted at 37°C for a shorter duration of 5 h. Subsequently, the jars containing the fermented samples were then cooled, transferred to a refrigerator, and stored at 6°C for 35 days. Analysis of the yogurt-type fermented soy beverage was performed at specific intervals, namely 0, 7, 14, 21, 28, and 35 days.

### 2.3. Determination of pH changes

The pH changes occurring during fermentation were monitored using a CPO-505 pH meter (Elmetron, Zabrze, Poland). To standardize the glass electrode, two buffers (pH 7.0 and pH 4.0) were used. These buffers were disinfected with a 70% v/v alcoholic solution and rinsed with sterilized distilled water before each measurement. The pH was automatically measured at intervals of 60 min. The pH readings were recorded with a precision of 0.01 units. The presented data represents the average values obtained from five independent replications.

The maximum acidification rate (V_max_) was calculated according to (Shiby and Mishra, 2008) using equation (1):


(1) $$V_{\max}={\left(\frac{\Delta \mathrm{pH}}{\Delta t}\right)}_{\max}$$

where: V_max_ is the maximum acidification rate; ∆pH is the difference in pH during time; ∆t is the time for which the pH change was calculated.

and expressed in absolute values. The time at which the maximum acidification rate was obtained (T_max_) and the time at which pH 4.5 was reached (T_e_) were considered responses that characterized the kinetics of the process. The data presented are averages of five independent replications.

The pH changes during refrigerated storage of yogurt-type fermented soy beverage were measured following the methodology described in the study by Ziarno et al. (2020). The pH meter electrode was immersed in the sample, and the reading was made with an accuracy of 0.01. The presented data represents the average values obtained from five independent replications.

### 2.4. Determination of live cell population of starter microflora during refrigerated storage of yogurt-type fermented soy beverage

The microbiological evaluation involved determining the population of microbial cells used as a starter by employing the plate method described in a previous study by Ziarno et al. (2019). To assess the counts of lactobacilli and bifidobacteria, De Man, Rogosa and Sharpe (MRS) agar (Merck, Darmstadt, Germany) supplemented with L-cysteine (0.05 g of L-cysteine/100 mL of MRS) was utilized. Streptococci counts were determined using M17 agar (Merck, Darmstadt, Germany). The inoculated plates were then incubated at 37°C for 72 h, under either anaerobic conditions (for lactobacilli and bifidobacteria) or aerobic conditions (for streptococci). Following incubation, all colonies were counted, and the average result was reported as the logarithm of colony-forming units per gram (log CFU/g). The data presented represent the average values obtained from five independent replications.

### 2.5. Water-holding capacity (WHC) during refrigerated storage of yogurt-type fermented soy beverage

The water-holding capacity (WHC) of the yogurt-type fermented soy beverage was assessed following the methodology outlined in the study by Ziarno et al. (2019). Briefly, 40 g of the sample was carefully weighed into a 50 mL Falcon tube. The tube was then subjected to centrifugation at a speed of 16,128 × g, maintained at 4°C for 20 min. The expelled liquid was subsequently removed and weighed. The remaining residue (referred to as M1) was utilized to determine the water retention of each sample. The test was conducted using equation (2) as provided below:


(2) $$WHC\;\left[\%\right]=\left(\frac{M1}{M2}\right)\times 100$$

where: M1 is the mass of precipitate after centrifugation (in grams) and M2 is the mass of each beverage sample (in grams). The data presented are averages of five independent replications.

### 2.6. Texture analysis during refrigerated storage of yogurt-type fermented soy beverage

Texture analysis, specifically the assessment of hardness and adhesiveness properties, was performed on the fermented samples using the Brookfield CT3 Texture Analyzer (Brookfield Engineering Laboratories, Middleborough, Massachusetts, United States). The analysis was conducted following the method outlined in the study by Kycia et al. (2018). The measurement was made with a cylindrical probe TA4/1000 (diameter 38.1 mm and height 20 mm). A pressure force of 0.04 N was applied during the measurement. During the measurement, the probe used was moved toward the inside of the sample at a speed of 2 mm/s and in the opposite direction when withdrawing from the sample at 4.5 mm/s. Hardness was expressed in N units and adhesiveness in mJ. The results were analyzed using the TexturePro CT V1.4 Build 17 software included in the measurement kit.

### 2.7. Analysis of carbohydrates content during refrigerated storage of yogurt-type fermented soy beverage

The carbohydrate content was measured utilizing high-performance liquid chromatography (HPLC) with a Sykam instrument (Fürstenfeldbruck, Germany). Sample preparation and chromatographic analysis were performed following the procedures described in the study by Ziarno et al. (2019). Briefly, 8 g of each sample was homogenized with 32 g of methanol (HPLC grade; Sigma-Aldrich, St. Louis, Missouri, United States) using an automatic shaker and an ultrasonic bath (for 30 min). The samples were then centrifuged (16,000× *g*, 4°C, 30 min) and filtered through syringe filter (0.22 μm). The analysis was carried out with a guard column Sugar-D (10 mm × 4.6 mm, 5 μm; Cosmosil, Nacalai Tesque, Kyoto, Japan) and column Sugar-D (250 mm × 4.6 mm, 5 μm; Cosmosil). The chromatographic separation parameters were as follows: flow 1 mL/min, oven temperature 30°C, range of detector 10,000 mV, and sample rate 2 Hz. The mobile phase was a 60: 40 mixture of acetonitrile (HPLC-grade, Sigma-Aldrich) and deionized water. Carbohydrate concentration was calculated based on the standard curves (of fructose, galactose, glucose, sucrose, raffinose, stachyose, and verbascose; HPLC grade; Sigma-Aldrich, St. Louis, Missouri, United States) and expressed as milligrams of sugar per 100 g samples (mg%). The presented data represents the average values obtained from five independent replications.

### 2.8. Determination of fatty acid profile during refrigerated storage of yogurt-type fermented soy beverage

The fatty acid profile of the yogurt-type fermented soy beverage samples was analyzed using gas chromatography combined with mass spectrometry (GC–MS) with a Shimadzu GC–MS Q2010 instrument (Kyoto, Japan). Sample preparation and chromatographic analysis for determining the fatty acid profile followed the methodology described in the study by Derewiaka et al. (2011). Briefly, for the extraction of the lipid fraction from cheese samples, the Folch method was used and prior to analysis, the analyzed fat samples were converted to fatty acid methyl esters. The analytical methodology employed was under the procedures outlined in the study by Ziarno et al. (2020). Briefly, the fatty acid profiling chromatography column was a BPX 70 packing (30 m length, 0.25 mm internal diameter, 0.25 μm film thickness, Shim-Pol A. M. Borzymowski, Warsaw, Poland). During the measurement, the temperature profile increased from an initial 60°C and gradually increased by 10°C/min until it reached 180°C. The temperature then jumped to 230°C with an increase of 3°C/min., and the set temperature was maintained for 15 min. Injector and detector temperatures were maintained at 225°C and 250°C, respectively. Available standards and the areas of the peaks in the chromatograms were used to determine the fatty acid profile. The presented data represents the average values obtained from five independent replications.

To evaluate the nutritional quality of the yogurt-type fermented soy beverage samples, two indices were calculated: the atherogenic index (AI), which indicates the propensity to develop microcoronary and macrocoronary diseases, and the thrombogenic index (TI), which reflects the likelihood of clot formation in blood vessels. The calculations were calculated using equations (3) and (4) as outlined in the studies (Ghaeni et al., 2013; Šimat et al., 2015; Hashempour-Baltork et al., 2018; Pena-Serna et al., 2019) as follow:


(3) $$\mathrm{AI}=\frac{C\;12:0+\left(4\times C\;14:0\right)+C\;16:0}{\mathrm{MUFA}+\mathrm{PUFA}}$$

(4) $$\mathrm{TI}=\frac{C\;14:0+C\;16:0+C\;18:0}{\begin{array}{l} 0.5\times \mathrm{MUFA}+0.5\times \mathrm{PUFA}\;n-6+\left(3\times \mathrm{PUFA}\;n-3\right)+\left(\frac{\mathrm{PUFA}\;n-3}{\mathrm{PUFA}\;n-6}\right) \end{array}}$$

where: MUFA are monounsaturated fatty acids, PUFA are polyunsaturated fatty acids.

### 2.9. Statistical analysis

The experiments were conducted following a completely randomized design, and the data obtained were subjected to statistical analysis using analysis of variance (ANOVA). The ANOVA was utilized to determine significant differences in the mean values of the evaluated parameters among the fermented beverages. Tukey’s comparison test was employed to identify differences between the means obtained from the ANOVA. A significance level of *p* < 0.05 was considered to determine statistical significance. The software Statgraphics 18 Centurion (Statgraphics Technologies, The Plains, Virginia, United States) was utilized for the statistical analysis.

## 3. Results and discussion

### 3.1. Acidification curves and acidification kinetics

**Figure 1:**
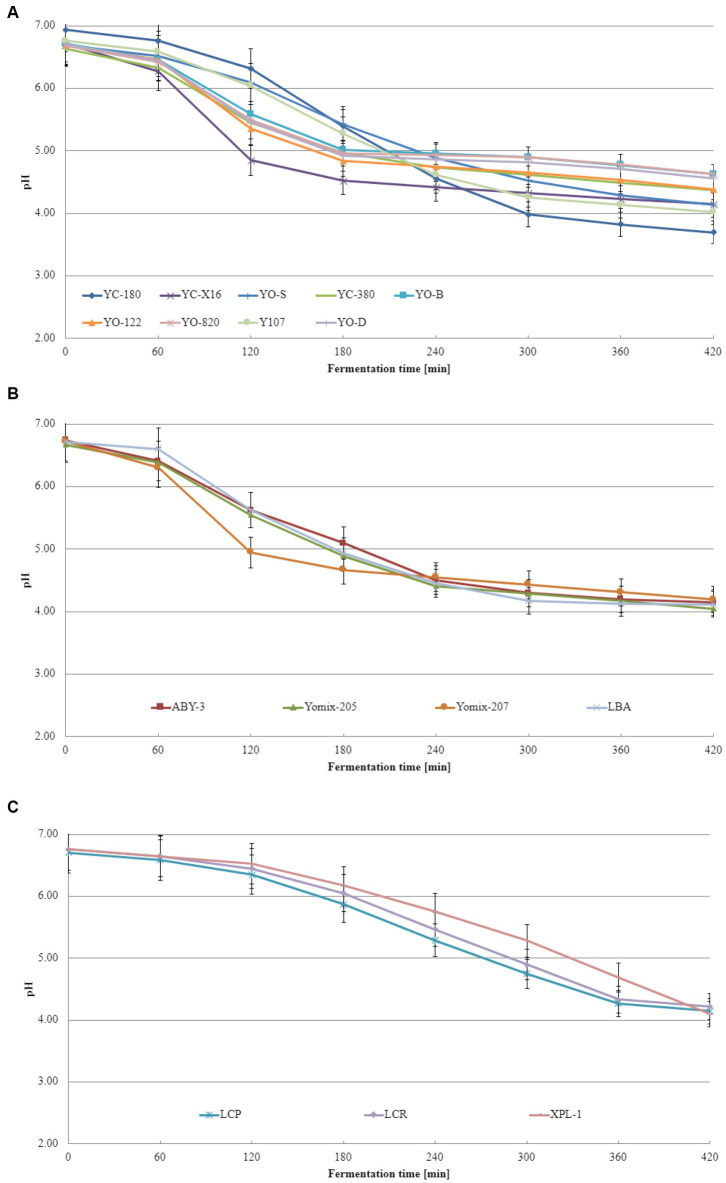
Acidification curves of the base soy beverage with different dairy starter cultures: **(A)** dairy starter cultures consisted of typical yogurt microorganisms comprising *L. delbrueckii* species and *S. thermophilus* species, **(B)** dairy starter cultures contained not only *L. delbrueckii* species and *S. thermophilus* but also additional microflora, and **(C)** dairy starter cultures consisted of monocultures of lactobacilli or a mixture of different lactic acid bacteria (means and standard deviations). Tukey's Honestly Significant Difference (HSD) = 0.05.

Acidification curves depict the pH changes that occur during the lactic acid fermentation process. These curves illustrate how pH evolves as fermentation progresses, starting from the initial state and culminating in the final pH. By examining acidification curves, one can determine the duration required to achieve the desired level of acidity, which is crucial for ensuring consistent product quality. Additionally, these curves exhibit distinct characteristics based on the lactic bacteria species involved. Acidification curves refer to the graphical representation of pH variations throughout a fermentation process, providing a visual depiction of how acidity changes over time. This curve offers valuable insights into the rate at which acid is produced, the time needed to attain specific pH levels and the overall efficiency of the fermentation process. Figure 1 displays the acidification curves obtained from these experiments, with Figure 1A showcasing the curves for dairy starter cultures consisted of typical yogurt microorganisms comprising *L. delbrueckii* species and *S. thermophilus* species. It is evident that different dairy starter cultures exhibit varying pH changes during fermentation, and these discrepancies are statistically significant across cultures (the calculated value of Tukey’s Honestly Significant Difference represents the minimum difference between the means that is considered statistically significant at the assumed level of significance). The dairy starter cultures contained not only *L. delbrueckii* species and *S. thermophilus* but also additional microflora (Figure 1B) display similar acidification curves, but statistical differences were observed between them as well. Figure 1C showcases a distinct trajectory for the acidification curves of other dairy starter cultures. In their case, the acidification rate was the slowest. The explanation for this phenomenon could be that these starter cultures contained monocultures of lactobacilli or a mixture of different lactic acid bacteria, including lactobacillus spp., *Leuconostoc* spp., *Lactococcus* spp., etc. for which the fermentation conditions used were not optimal. It is worth noting that the recommended fermentation temperature for XPL-1 culture is in the range of 30–35°C and using a higher temperature may explain the T_e_ value obtained.

**Figure 2:**
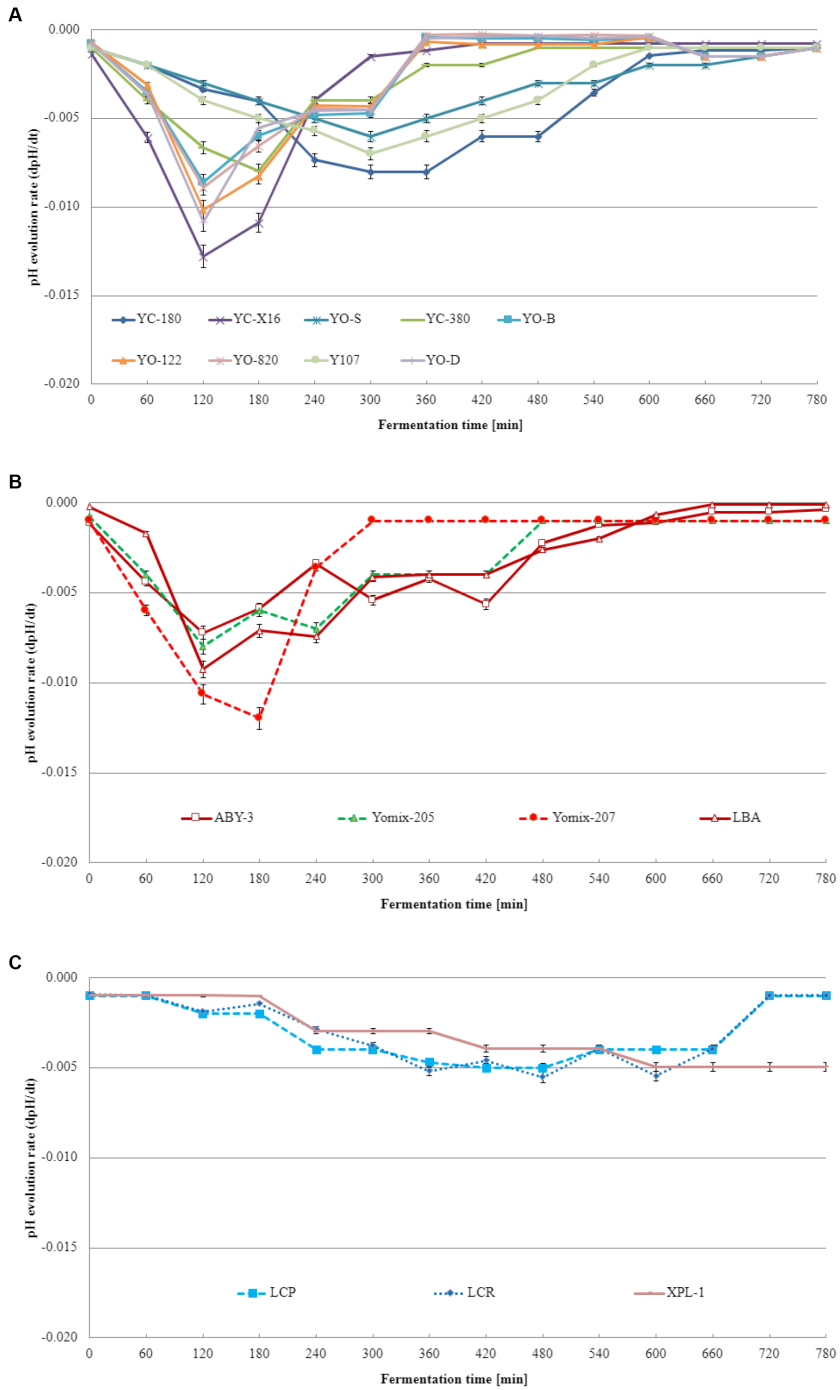
Acidification rate of the base soy beverage with different dairy starter cultures: **(A)** dairy starter cultures consisted of typical yogurt microorganisms comprising *L. delbrueckii* species and *S. thermophilus* species, **(B)** dairy starter cultures contained not only *L. delbrueckii* species and *S. thermophilus* but also additional microflora, and **(C)** dairy starter cultures consisted of monocultures of lactobacilli or a mixture of different lactic acid bacteria (means and standard deviations). Tukey's Honestly Significant Difference (HSD) = 0.05.

Acidification curves visually represent the pH changes during fermentation, while acidification kinetics involves mathematical models that describe the kinetics of the process. Acidification kinetics encompass the mathematical models that depict pH changes over time in fermentation. These models consider various variables, such as initial pH, temperature, substrate, and microorganism concentrations, influencing the fermentation process. Acidification kinetics enables the prediction of acid production rate and final pH under different conditions. The acidification curves obtained from these experiments are displayed in Figure 2. Notably, acidification curves differ among dairy starter cultures consisted of typical yogurt microorganisms (Figure 2A), dairy starter cultures contained not only *L. delbrueckii* species and *S. thermophilus* but also additional microflora (Figure 2B), and other dairy starter cultures (Figure 2C). For dairy starter cultures consisted of typical yogurt microorganisms, the maximum acidification rate ranged from 0.006 to 0.013. These differences were statistically significant. In case of starter cultures contained not only *L. delbrueckii* species and *S. thermophilus* but also additional microflora, the maximum acidification rate varied from 0.007 to 0.012 depending on the starter culture tested. Other starter cultures (consisted of monocultures of lactobacilli or a mixture of different lactic acid bacteria) exhibited a maximum acidification rate ranging from 0.005 to 0.006. Acidification kinetics play a pivotal role in distinguishing dairy starter cultures. Different dairy starter cultures exhibit varying rates of milk acidification, influencing the final product’s quality. Acidification kinetics are influenced by multiple factors, including temperature, pH, bacterial concentration, and beverage composition. Consequently, distinct lactic acid bacteria cultures display characteristic acidification patterns and differ in their acidification kinetics. These differences in acidification kinetics allow for the differentiation and selection of dairy starter cultures best suited for specific product production.

To the best of our knowledge and extensive literature search across various scientific databases, no studies have been conducted on the fermentation kinetics of soy beverages using dairy starter cultures intended for yogurt production. However, Bezerra et al. (2012) revealed that the acidification of milk-based yogurts exhibited Vmax values ranging from 15.1 to 18.9 pH units/min, T_max_ values ranging from 179 to 210 min, and T_e_ values ranging from 260 to 267 min. Similar values were obtained in this study for soy beverages fermented with specific dairy starter cultures. These findings align with those reported by Fawzi et al. (2022) for rice-based yogurt. Although acidification curves and kinetics of the base soy beverage have been extensively studied in this research, the literature lacks information on the acidification kinetics of the base soy beverage with dairy starter cultures consisted of typical yogurt microorganisms (*L. delbrueckii* species and *S. thermophilus*), dairy starter cultures contained not only typical yogurt microorganisms but also additional microflora, or dairy starter cultures consisted of monocultures of lactobacilli or a mixture of different lactic acid bacteria. Based on the obtained acidification curve and kinetics data, two dairy starter cultures were identified as the most suitable for fermenting the base soy beverage: YC-X16 consisted of typical yogurt microorganisms comprising *L. delbrueckii* species and *S. thermophilus* species culture and YOMIX-207 contained not only typical yogurt microorganisms but also additional microflora.

### 3.2. Starter microflora population during refrigerated storage of yogurt-type fermented soy beverage

The starter cultures have been extensively utilized in the dairy industry for a considerable period and have undergone comprehensive research to validate their efficacy and stability. Their utilization instills confidence in the success of the fermentation process and ensures the desired quality characteristics of soy beverages. Dairy starter cultures often present a cost advantage compared to vegan starter cultures. Their wide adoption in the dairy industry allows for economies of scale, resulting in reduced costs. Assessing the live cell populations following the application of dairy starter cultures for yogurt production is crucial in achieving a safe and high-quality product. Statistical analysis revealed that the choice of dairy starter cultures significantly influenced the initial number of live cells in the starter microflora, both for the overall lactobacilli and bifidobacteria populations (bifidobacteria were present only in some starter cultures tested), as well as the streptococci and lactococci populations (*Lactococcus* spp. and *Leuconostoc* spp. were present only in some starter cultures tested) population (Table 2). The duration of refrigeration storage of the fermented soy beverage samples emerged as another statistically significant factor affecting the live cell populations in the starter microflora. For soy beverages prepared using starters such as YC-380, YO-820, YO-D, YO-B, YO-S, YO-122, YC-180, YOMIX-205, YOMIX-207, and LBA, storage time induced statistically significant changes in the number of live cells of the starter microflora, impacting the total populations of lactobacilli and bifidobacteria, as well as the streptococci population. Conversely, beverages prepared with starters like YC-X16, Y107, ABY-3, XPL-1, and LCP did not exhibit statistically significant changes in the cell population of the starter microflora. This emphasizes the importance of selecting the appropriate starter culture to achieve high-quality yogurt-type fermented soy beverage suitable for consumption. Determining the correlation between the acidifying rate and the population of lactic acid bacteria would be valuable in determining the optimal starter culture for the production of a high-quality yogurt-type fermented soy beverages.

**Table 2:** Changes in starter microorganisms population during refrigerated storage of yogurt-type fermented soy beverage.

Ref.	Population of lactobacilli and bifidobacteria [log(CFU/g)]						Population of streptococci [log(CFU/g)]					
Storage time [day]	0	7	14	21	28	35	0	7	14	21	28	35
YC-X16	6.4 ± 0.3^a^	6.7 ± 0.3^a,b^	6.7 ± 0.4^a,b^	6.6 ± 0.3^a^	6.5 ± 0.2^a^	6.4 ± 0.3^a^	8.1 ± 0.2^e,f^	7.9 ± 0.3^e,f^	7.9 ± 0.3^e,f^	7.9 ± 0.2^e,f^	7.8 ± 0.3^e^	7.7 ± 0.3^e^
YC-380	6.5 ± 0.1^a^	6.7 ± 0.1^a,b^	6.9 ± 0.2^a,b^	7.0 ± 0.2^b,c^	7.1 ± 0.2^b,c^	6.9 ± 0.1^a,b^	7.3 ± 0.2^c^	7.4 ± 0.2^c^	7.6 ± 0.2^e^	7.7 ± 0.2^e^	7.8 ± 0.1^e^	7.7 ± 0.2^e^
YO-820	7.2 ± 0.2^b^	7.3 ± 0.2^c,d^	7.4 ± 0.2^c,d^	7.5 ± 0.2^c,d^	7.3 ± 0.2^c,d^	7.2 ± 0.2^b,c^	8.0 ± 0.1^e,f^	8.1 ± 0.1^e,f^	8.2 ± 0.1^f,g^	8.3 ± 0.2^f,g^	8.1 ± 0.2^e,f^	8.0 ± 0.2^e,f^
Y107	7.5 ± 0.2^c,d^	7.5 ± 0.2^c,d^	7.5 ± 0.2^c,d^	7.4 ± 0.2^c,d^	7.4 ± 0.2^c,d^	7.4 ± 0.2^c,d^	8.2 ± 0.1^f,g^	8.2 ± 0.1^f,g^	8.2 ± 0.1^f,g^	8.1 ± 0.2^e,f^	8.1 ± 0.2^e,f^	8.2 ± 0.1^f,g^
YO-D	7.3 ± 0.2^c^	7.3 ± 0.2^c^	7.6 ± 0.2^d,e^	7.8 ± 0.2^d,e^	7.8 ± 0.3^d,e^	7.5 ± 0.3^c^	8.0 ± 0.2^e,f^	8.0 ± 0.2^e,f^	8.3 ± 0.2^f^	8.3 ± 0.3^f^	8.2 ± 0.1^f,g^	8.0 ± 0.1^e,f^
YO-B	6.5 ± 0.1^a^	6.6 ± 0.1^a^	6.9 ± 0.2^a^	7.2 ± 0.2^b,c^	7.4 ± 0.2^d,e^	7.2 ± 0.2^b,c^	7.3 ± 0.2^c^	7.4 ± 0.2^c^	7.6 ± 0.2^e^	7.9 ± 0.1^e,f^	8.2 ± 0.1^g^	7.9 ± 0.2^e,f^
YO-S	6.8 ± 0.2^a^	6.9 ± 0.2^a^	7.0 ± 0.2^b,c^	7.1 ± 0.2^b,c^	7.1 ± 0.2^b,c^	7.1 ± 0.2^b,c^	7.5 ± 0.1^c^	7.7 ± 0.2^e^	7.8 ± 0.1^e^	7.9 ± 0.1^e,f^	7.9 ± 0.2^e,f^	7.9 ± 0.1^e,f^
YO-122	6.9 ± 0.2^a^	7.0 ± 0.2^b,c^	7.1 ± 0.2^b,c^	7.2 ± 0.2^b,c^	7.0 ± 0.2^b,c^	6.9 ± 0.2^b^	7.6 ± 0.2^d^	7.7 ± 0.2^e^	7.8 ± 0.2^e^	7.9 ± 0.2^e,f^	7.7 ± 0.2^e^	7.6 ± 0.2^e^
YC-180	7.0 ± 0.2^b,c^	7.0 ± 0.2^b,c^	7.1 ± 0.2^b,c^	7.2 ± 0.2^b,c^	7.2 ± 0.2^b,c^	7.2 ± 0.2^b,c^	7.8 ± 0.2^d^	7.7 ± 0.2^e^	7.8 ± 0.2^e^	7.9 ± 0.2^e,f^	7.9 ± 0.2^e,f^	8.0 ± 0.2^e,f^
ABY-3	7.9 ± 0.2^e,f^	7.9 ± 0.2^e,f^	7.9 ± 0.2^e,f^	7.8 ± 0.1^d,e^	7.8 ± 0.2^d,e^	7.6 ± 0.2^e,f^	8.6 ± 0.2^g,h^	8.6 ± 0.2^g,h^	8.6 ± 0.2^g,h^	8.5 ± 0.2^g,h^	8.5 ± 0.2^g,h^	8.4 ± 0.2^f,g^
YOMIX-205	7.2 ± 0.2^b,c^	7.2 ± 0.3^b,c^	7.2 ± 0.2^b,c^	7.2 ± 0.2^b,c^	7.1 ± 0.2^b,c^	7.1 ± 0.2^b,c^	7.9 ± 0.2^e,f^	7.8 ± 0.2^e,f^	7.9 ± 0.2^e,f^	7.9 ± 0.2^e,f^	7.8 ± 0.2^e,f^	7.9 ± 0.2^e,f^
YOMIX-207	7.8 ± 0.2^d,e^	7.8 ± 0.3^d,e^	8.0 ± 0.2^e,f^	7.8 ± 0.2^d,e^	7.8 ± 0.2^d,e^	7.7 ± 0.1^d,e^	7.4 ± 0.2^c^	7.5 ± 0.3^c^	7.6 ± 0.2^e^	7.6 ± 0.3^d^	7.6 ± 0.2^d^	7.5 ± 0.2^c^
LBA	7.0 ± 0.2^b,c^	7.0 ± 0.1^b,c^	7.1 ± 0.2^b,c^	7.3 ± 0.2^c,b^	7.2 ± 0.2^b,c^	7.2 ± 0.1^b,c^	7.7 ± 0.2^e^	7.8 ± 0.1^e^	7.9 ± 0.1^e,f^	8.0 ± 0.2^e,f^	8.0 ± 0.2^e,f^	8.0 ± 0.1^e,f^
XDL-1	6.9 ± 0.2^b^	7.0 ± 0.2^b,c^	7.0 ± 0.2^b,c^	7.2 ± 0.2^b,c^	7.1 ± 0.2^b,c^	7.0 ± 0.1^b,c^	7.6 ± 0.1^e^	7.8 ± 0.2^e^	7.8 ± 0.1^e^	8.0 ± 0.1^e,f^	7.9 ± 0.2^e,f^	7.8 ± 0.2^e^
LCP	7.4 ± 0.2^c,d^	7.5 ± 0.2^c,d^	7.3 ± 0.2^c,d^	7.4 ± 0.2^c,d^	7.3 ± 0.2^c,d^	7.2 ± 0.2^b,c^	8.1 ± 0.2^e,f^	8.2 ± 0.2^f,g^	8.0 ± 0.2^e,f^	8.1 ± 0.2^e,f^	8.0 ± 0.2^e,f^	7.9 ± 0.2^e,f^
LCR	7.1 ± 0.1^b,c^	7.4 ± 0.1^c,d^	7.4 ± 0.2^c,d^	7.4 ± 0.2^c,d^	7.4 ± 0.2^d,e^	7.2 ± 0.2^b,c^	7.8 ± 0.3^d^	8.1 ± 0.2^e,f^	8.2 ± 0.1^f,g^	8.2 ± 0.2^f,g^	8.2 ± 0.2^g^	8.0 ± 0.1^e,f^

Different letters in the whole table indicate significant differences (*p* < 0.05).

Previous studies have demonstrated that the survival rate of lactobacilli and streptococci in plant-based beverages depends on the type of beverage and the specific starter culture used (Zaręba and Ziarno, 2017). In yogurt production, a common combination of lactic acid bacteria involves *S. thermophilus* and *L. delbrueckii* subsp. *bulgaricus*, which exhibits a synergistic relationship in milk fermentation. *S. thermophilus* exhibit rapid growth in milk and produce organic acids and carbon dioxide, which stimulates the growth of *L. delbrueckii* subsp. *bulgaricus*. However, *S. thermophilus* generally have low proteolytic activity, while *L. delbrueckii* subsp. *bulgaricus* exhibit higher proteolytic activity, resulting in the production of peptides and free amino acids that serve as a nitrogen source for *S. thermophilus* (Savijoki et al., 2006; Tian et al., 2018; Ji et al., 2021). The combination of these lactic acid bacteria has been utilized in the production of yogurt-type plant-based fermented beverages (Jiménez-Martínez et al., 2003; Grygorczyk and Corredig, 2013; Brückner-Gühmann et al., 2019; Klost and Drusch, 2019; Grasso et al., 2020; Laaksonen et al., 2021; Levy et al., 2021; Ogundipe et al., 2021; Dhakal et al., 2023). However, limited research has explored the functionality of these systems on the proteins of plant-based foods. Previous studies have mainly focused on soy as one of the most commonly used plant alternatives to dairy products (Donkor et al., 2007; Aguirre et al., 2008; Pescuma et al., 2013; Boulay et al., 2020; Shirotani et al., 2021). Studies by Chou and Hou (2000), Farnworth et al. (2007), and Tang et al. (2007) observed an increase in viable bacterial cell numbers of *S. thermophilus* and *L. delbrueckii* subsp. *bulgaricus* species in yogurt-type fermented soy beverage, with fermentation time varying depending on the bacterial strain used. Plant-based yogurt alternatives often employ *S. thermophilus*, *L. delbrueckii* subsp. *bulgaricus*, *Lactiplantibacillus plantarum*, and *Lactobacillus acidophilus* for fermentation (Michaylova et al., 2007; Boeck et al., 2021; Montemurro et al., 2021). Additionally, there are scientific reports highlighting the ability of bifidobacteria strains to thrive in soy beverages (due to the ability to utilize soy oligosaccharides), particularly in the presence of lactic acid or propionic acid microflora (Kamaly, 1997; Wu et al., 2012; Delgado et al., 2019). In the present study, it was observed that the population of typical yogurt microorganisms decreased during refrigerated storage of fermented soy beverage samples, with a reduction depending on the specific starter culture used. Conversely, some researchers have reported an initial increase in bacterial population during the first few days of refrigerated storage, followed by a subsequent reduction (Beasley et al., 2002). The survival rate of starter culture-derived bacteria is indeed a critical factor in producing a high-quality and healthy fermented product. Maintaining a high population level of live cells of microorganisms throughout the shelf life is indicative of the product’s quality and its potential health benefits. According to the FAO/WHO guidelines, yogurt-type products should have a minimum cell count of at least 7 log CFU/mL or g for yogurt bacteria and at least 6 log CFU/mL or g for accessory microorganisms, including probiotic strains (FAO/WHO, 2001). In the study mentioned, all tested dairy starter cultures demonstrated excellent survival of yogurt microflora during the 35-day refrigerated storage of fermented soy beverage samples.

### 3.3. The pH value changes during refrigerated storage of yogurt-type fermented soy beverage

Changes in pH values during refrigerated storage of yogurt-type fermented soy beverage are crucial indicators of product stability and quality over time. The pH value plays a crucial role in preserving the beverage and influencing its sensory characteristics. Initially, the pH values of the freshly obtained yogurt-type fermented soy beverage were statistically significantly influenced by the type of culture starter used (Table 3). During refrigerated storage, the pH values of the yogurt-type fermented soy beverage decreased, but the extent of this change also depended on the type of starter used (D'Alessandro et al., 2023). After 35 days of refrigerated storage, the pH value of the samples demonstrated a statistically significant reduction compared to the pH values of the fresh samples. It can also be noted that between 21 and 35 days of refrigerated storage of the samples, the pH changes become less pronounced compared to the initial 2–3 weeks. This may indicate a stabilization of the acidity in the fermented soy beverage samples.

**Table 3:** pH value changes during refrigerated storage of yogurt-type fermented soy beverage.

Storage time [day] Ref.	0	7	14	21	28	35
YC-X16	4.62 ± 0.06^d^	4.53 ± 0.21^c^	4.51 ± 0.15^b,c^	4.51 ± 0.15^b,c^	4.48 ± 0.10^b^	4.43 ± 0.16^b^
YC-380	4.88 ± 0.07^f^	4.69 ± 0.32^d^	4.50 ± 0.21^b^	4.50 ± 0.19^b^	4.38 ± 0.13^a^	4.31 ± 0.05^a^
YO-820	4.51 ± 0.11^b,c^	4.64 ± 0.11^d^	4.36 ± 0.10^a^	4.40 ± 0.05^a^	4.38 ± 0.15^a^	4.41 ± 0.15^a,b^
Y107	4.78 ± 0.10^e^	4.74 ± 0.10^e^	4.60 ± 0.10^c^	4.61 ± 0.10^c,d^	4.61 ± 0.10^c,d^	4.68 ± 0.10^d^
YO-D	4.73 ± 0.10^e^	4.60 ± 0.10^c^	4.46 ± 0.10^b^	4.45 ± 0.10^b^	4.45 ± 0.12^b^	4.52 ± 0.10^c^
YO-S	4.91 ± 0.02^f,g^	4.54 ± 0.08^c^	4.50 ± 0.10^b^	4.47 ± 0.10^b^	4.37 ± 0.10^a^	4.42 ± 0.10^b^
YO-B	4.82 ± 0.11^f^	4.65 ± 0.10^d^	4.42 ± 0.10^b^	4.43 ± 0.10^b^	4.46 ± 0.10^b^	4.49 ± 0.10^b^
YO-122	4.88 ± 0.09^f^	4.69 ± 0.18^d^	4.73 ± 0.15^e^	4.80 ± 0.12^e^	4.77 ± 0.13^e^	4.83 ± 0.20^f^
YC-180	4.88 ± 0.06^f^	4.68 ± 0.08^d^	4.56 ± 0.10^c^	4.54 ± 0.09^c^	4.57 ± 0.10^c^	4.53 ± 0.08^c^
ABY-3	4.90 ± 0.04^f^	4.67 ± 0.06^d^	4.59 ± 0.00^c^	4.53 ± 0.00^c^	4.35 ± 0.10^a^	4.36 ± 0.09^a^
YOMIX-205	4.90 ± 0.04^f^	4.63 ± 0.02^d^	4.52 ± 0.13^c^	4.49 ± 0.13^b^	4.46 ± 0.13^b^	4.46 ± 0.13^b^
YOMIX-207	4.70 ± 0.12^d^	4.54 ± 0.09^c^	4.60 ± 0.01^c^	4.57 ± 0.08^c^	4.52 ± 0.05^c^	4.49 ± 0.11^b^
LBA	4.81 ± 0.11^f^	4.62 ± 0.10^d^	4.46 ± 0.10^b^	4.55 ± 0.10^c^	4.51 ± 0.10^b,c^	4.57 ± 0.10^c^
XDL-1	4.84 ± 0.11^f^	4.77 ± 0.11^e^	4.60 ± 0.10^c^	4.42 ± 0.10^b^	4.43 ± 0.10^b^	4.41 ± 0.10^a,b^
LCP	5.06 ± 0.16^h^	5.01 ± 0.26^g,h^	4.77 ± 0.11^e^	4.68 ± 0.14^d^	4.62 ± 0.16^d^	4.64 ± 0.15^d^
LCR	4.58 ± 0.10^c^	4.50 ± 0.09^b^	4.43 ± 0.09^b^	4.40 ± 0.09^a^	4.43 ± 0.09^b^	4.44 ± 0.09^b^

Different letters in the whole table indicate significant differences (*p* < 0.05).

Monitoring pH changes during storage provides insights into the ongoing fermentation processes and the activity of microorganisms present in the beverage, helping to assess the microbial stability and shelf life of the product. Post-fermentation acidification, an undesirable process in fermented products, refers to continuous acidification beyond the optimal range due to persistent metabolic activity of the product’s microflora during its shelf-life (Deshwal et al., 2021). Information on the post-acidification kinetics of dairy starter cultures used in our experiences in not known. Earlier studies (Beasley et al., 2002; Zaręba et al., 2008; Li et al., 2014; Mondragón-Bernal et al., 2017; Zaręba and Ziarno, 2017; D'Alessandro et al., 2023) have already observed a reduction in the pH value of fermented soya beverages during refrigerated storage. These pH changes in yogurt-type fermented soy beverage reflect the ongoing lactic acid fermentation by lactic acid bacteria, which contributes to the tangy taste and preservation of the beverage. Low pH values indicate a high stabilization of the acidifying activity of the microflora present and also affect the viability of lactobacilli and streptococci. However, Bianchi et al. (2015) also observed a decrease in the pH of a fermented beverage made from quinoa and soy during 28 days of storage at 5°C, but the viability of the probiotic bacteria was maintained. Therefore, monitoring pH changes during refrigerated storage of yogurt-type fermented soy beverage is crucial to ensure product quality and identify potential issues related to microbial activity and spoilage. It enables producers to make informed decisions regarding product formulation, storage conditions, and shelf-life estimation.

### 3.4. Water-holding capacity during refrigerated storage of yogurt-type fermented soy beverage

WHC refers to a food product’s ability to retain water during production, storage, and consumption. In the context of yogurt-type fermented beverages, WHC plays a crucial role in determining product quality. A higher WHC value results in a creamier and smoother texture. The WHC is directly related to the amount of water the proteins can hold within the fermented beverage’s structure, influencing its density, texture, stability, and shelf life. Insufficient WHC can lead to water release during storage, causing changes in texture and flavor. Consumers generally prefer products with optimal WHC, avoiding a powdery or watery consistency. Hence, maintaining appropriate WHC levels is critical for fermented beverage manufacturers. In this study, yogurt-type fermented soy beverage made on the basis of industrial soy beverages and selected dairy starter cultures available for the production of yogurts, were examined. The samples displayed varying WHC values, which were significantly dependent on the type of starter culture used and the duration of refrigerated storage (Table 4). These differences were expected since the dairy starter cultures tested declared distinct viscosity properties. Samples fermented with YC-X16, YC-380, YO-820, YO-122, LBA, and XPL-1 cultures exhibited relatively stable water-holding capacities throughout the 35-day refrigerated storage period. Among these, the yogurt-type fermented soy beverage utilizing the YC-X16 culture had the highest initial WHC value, aligning with the manufacturer’s claim of high viscosity in milk. In contrast, samples fermented with YO-D, YO-S, YO-B, YC-180, ABY-3, YOMIX-205, YOMIX-207, and LCR cultures experienced a statistically significant reduction in WHC compared to freshly obtained yogurt-type fermented soy beverage. Notably, among this group, the yogurt-type fermented soy beverage using the YOMIX-207 culture, known for their high viscosity properties (declared by the manufacturer in relation to milk fermentation), initially exhibited the highest WHC value. Conversely, samples fermented with Y107 and LCP cultures showed a statistically significant increase in final WHC values (averaging 32.55% ± 2.14 and 24.45% ± 4.78, respectively) compared to freshly obtained yogurt-type fermented soy beverage. Remarkably, the Y107 culture, also touted for its high viscosity in milk, demonstrated this effect.

**Table 4:** The WHC [%] value changes during refrigerated storage of yogurt-type fermented soy beverage.

Storage time [day] Ref.	0	7	14	21	28	35
YC-X16	34.30 ± 5.92^h,i^	33.09 ± 5.61^g,h^	35.22 ± 6.16^i^	34.92 ± 6.08^h,i^	32.65 ± 4.90^g,h^	32.94 ± 6.00^g,h^
YC-380	26.99 ± 1.00^d,e^	27.36 ± 1.01^e^	27.87 ± 1.03^e^	27.74 ± 1.03^e^	28.18 ± 1.04^e,f^	28.46 ± 1.05^e,f^
YO-820	26.90 ± 1.38^d,e^	26.22 ± 1.18^d,e^	26.31 ± 2.01^d,e^	25.25 ± 0.99^d^	27.19 ± 1.38^e^	27.26 ± 1.80^e^
Y107	29.94 ± 1.52^f^	32.42 ± 1.71^g,h^	31.53 ± 1.62^g^	31.40 ± 1.71^g^	33.20 ± 1.68^h^	32.55 ± 2.14^g,h^
YO-D	24.95 ± 1.51^c,d^	21.94 ± 2.96^b^	25.82 ± 2.60^d^	21.91 ± 1.53^b^	19.44 ± 1.24^a^	18.04 ± 1.16^a^
YO-S	30.25 ± 8.52^g^	28.07 ± 7.91^e,f^	28.07 ± 7.91^e,f^	30.41 ± 8.57^f,g^	29.31 ± 8.26^f^	28.22 ± 7.95^e,f^
YO-B	25.02 ± 1.57^c,d^	26.81 ± 1.67^d,e^	22.26 ± 1.43^b,c^	20.80 ± 1.35^b^	19.50 ± 1.28^a^	18.10 ± 1.21^a^
YO-122	25.02 ± 1.11^c,d^	23.96 ± 1.07^c^	23.87 ± 1.08^c^	23.66 ± 1.07^c^	23.25 ± 1.04^c^	22.54 ± 1.02^b,c^
YC-180	29.49 ± 6.32^f^	29.08 ± 5.78^e,f^	28.14 ± 5.46^e,f^	27.92 ± 5.40^e^	27.57 ± 5.40^e^	26.22 ± 5.55^d,e^
ABY-3	28.18 ± 5.54^e,f^	27.74 ± 5.22^e^	27.24 ± 5.06^e^	26.72 ± 4.93^d,e^	26.58 ± 4.94^d,e^	25.90 ± 5.01^d^
YOMIX-205	33.30 ± 4.57^h^	35.80 ± 5.17^i^	33.05 ± 4.52^g,h^	32.05 ± 4.31^g,h^	31.05 ± 4.12^f,g^	30.04 ± 3.96^f,g^
YOMIX-207	39.04 ± 3.32^j,k^	40.37 ± 3.53^k^	36.85 ± 3.69^i,j^	35.58 ± 3.99^i^	34.02 ± 3.74^h,i^	35.04 ± 4.98^h,i^
LBA	29.90 ± 2.51^e,f^	28.19 ± 2.39^e,f^	27.90 ± 2.34^e^	28.43 ± 2.39^e,f^	28.12 ± 2.36^e,f^	27.94 ± 2.34^e^
XDL-1	32.27 ± 8.03^g,h^	31.49 ± 7.81^g^	31.97 ± 7.95^g^	34.48 ± 8.69^h,i^	34.33 ± 8.64^h,i^	33.43 ± 8.16^h^
LCP	21.13 ± 4.30^a,b^	28.15 ± 5.31^e,f^	27.86 ± 5.27^e^	26.68 ± 5.10^d,e^	25.52 ± 4.93^d^	24.45 ± 4.78^c^
LCR	25.01 ± 1.27^c,d^	26.80 ± 1.35^d,e^	22.26 ± 1.16^b,c^	20.80 ± 1.10^a,b^	19.50 ± 1.04^a^	18.09 ± 0.98^a^

Different letters in the whole table indicate significant differences (*p* < 0.05).

The results of WHC during storage indicate potential syneresis, which refers to the loss of water from the product and can impact consistency and quality. The low WHC of yogurt-type fermented soy beverage limits consumer acceptance (Liu et al., 2023). Consistent with the present study, Xu et al. (2022) and Liu et al. (2023) corroborate the different WHC values observed in soy beverage samples fermented with different strains of lactic acid bacteria. These researchers attribute these differences to the varying ability of lactic acid bacteria to hydrolyze different protein subunits, influencing particle size distribution, zeta potential, and intermolecular forces in the soybean gel. The microstructure and texture of the product depend on these factors. Furthermore, the cited researchers also highlight the scarcity of information in the scientific literature concerning the impact of lactic acid bacteria on the textural characteristics of yogurt-type fermented soy beverages, particularly WHC. Xu et al. (2022) demonstrate that the WHC value of yogurt-type fermented soy beverages is closely linked to the internal structure of the gel network. The rapid metabolic activity of the starter culture bacteria leads to a quick decrease in pH value, reaching the isoelectric point of soy protein and forming a three-dimensional gel network composed of stable protein micelles. This structure results in a high WHC value, indicating that soy protein gels with well-developed networks have a greater WHC (Kovalenko and Briggs, 2002; Xu et al., 2022). However, excessive fermentation can lead to a loose protein network structure, resulting in a low WHC value (Xu et al., 2022). The type of microorganisms present in the dairy starter cultures, capable of producing exopolysaccharides (EPS), is the second crucial factor influencing water binding capacity (Duboc and Mollet, 2001; Li et al., 2014; Xu et al., 2019). In the present study, various dairy starter cultures tested claimed to possess high viscosity properties. However, since these claims were based on dairy beverage samples, the results obtained in this study for soy beverage samples did not always align with the declarations of the starter culture manufacturers.

### 3.5. Texture analysis during refrigerated storage of yogurt-type fermented soy beverage

**Figure 3:**
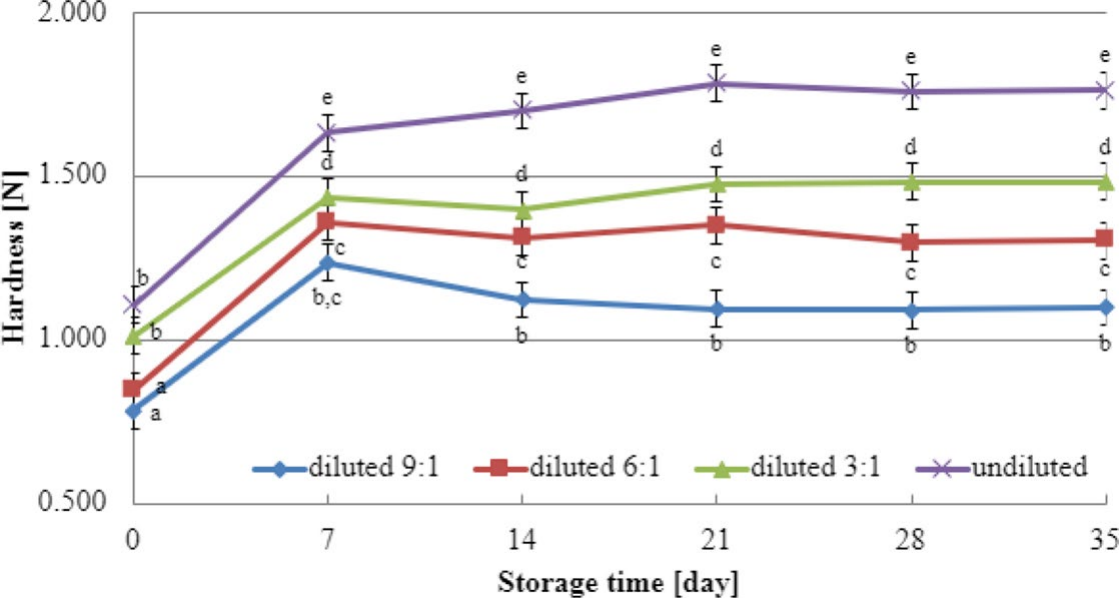
Changes in hardness value during refrigerated storage of soy beverages fermented by YOMIX-207 starter culture (means and honestly significant difference). Different letters in the same row indicate significant differences (*p* < 0.05).

**Figure 4:**
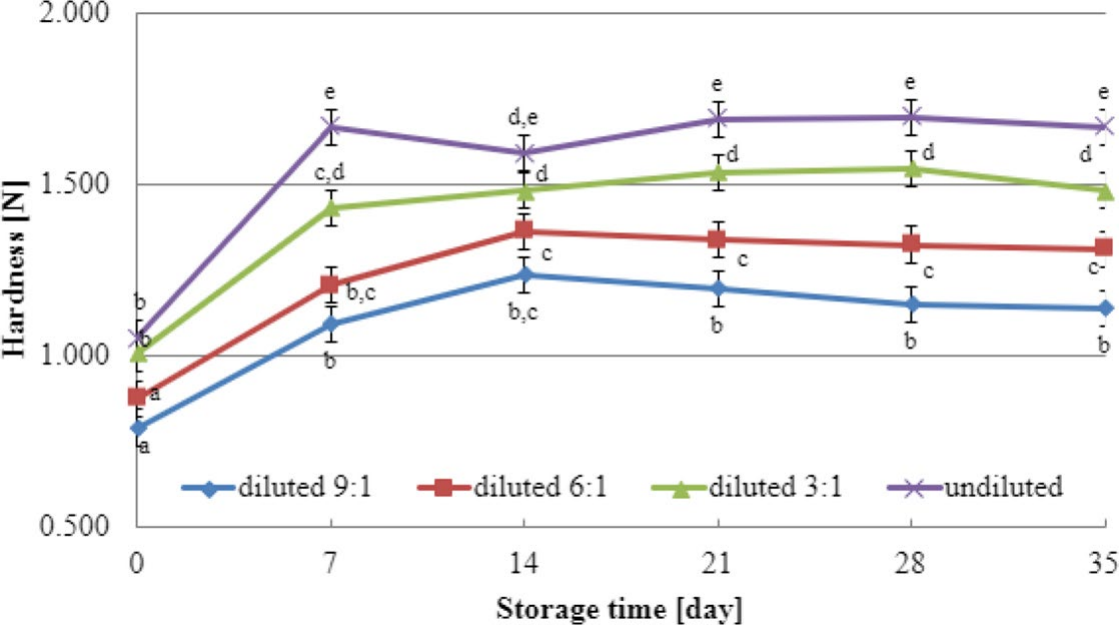
Changes in hardness value during refrigerated storage of soy beverages fermented by YC-X16 starter culture (means and honestly significant difference). Different letters in the same row indicate significant differences (*p* < 0.05).

Texture analysis results during storage can provide valuable insights into selecting the most suitable dairy culture for producing yogurt-type fermented soy beverages. In this study, texture testing was conducted on yogurt-type fermented soy beverage samples using two selected dairy starter cultures (YOMIX-207 and YC-X16). Considering the significance of dry matter content for texture parameters, the experiments compared the hardness and adhesiveness of beverages made from the base soy beverage diluted with different ratios of water: 3:1, 6:1, 9:1, and undiluted for comparison purposes (Figures 3, 4). The determination of hardness during the refrigerated storage period revealed a significant influence on texture analysis parameters for both YOMIX-207 and YC-X16 fermented soy beverage samples. Notably, the measured hardness values of yogurt-type fermented soy beverage remained relatively constant between 7 and 21 days of refrigerated storage.

**Figure 5:**
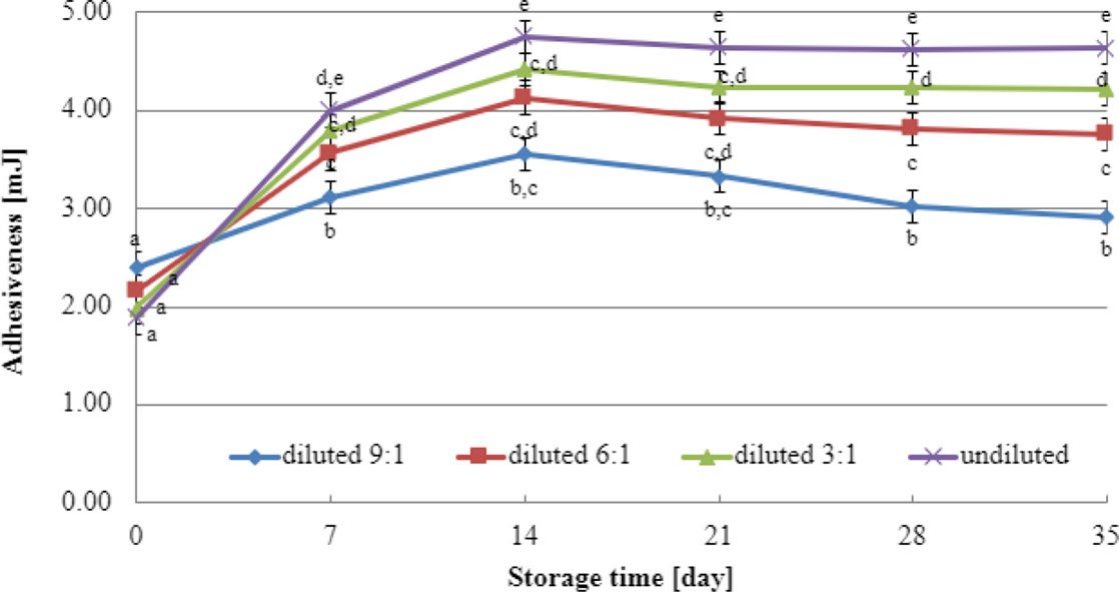
Changes in adhesiveness value during refrigerated storage of soy beverages fermented by YOMIX-207 starter culture (means and honestly significant difference). Different letters in the same row indicate significant differences (*p* < 0.05).

**Figure 6:**
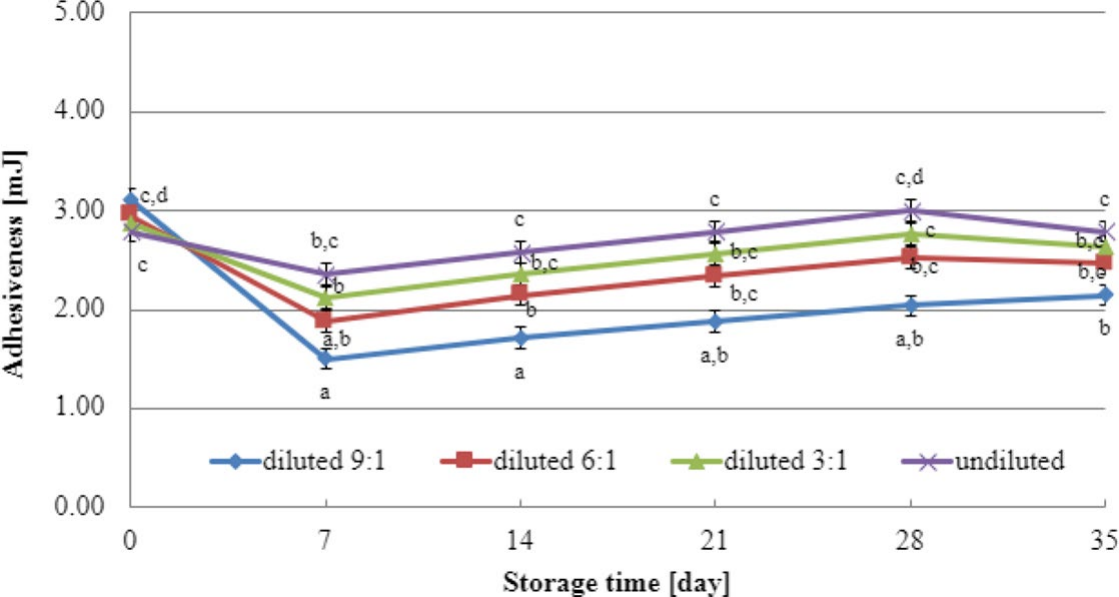
Changes in adhesiveness value during refrigerated storage of soy beverages fermented by YC-X16 starter culture (means and honestly significant difference). Different letters in the same row indicate significant differences (*p* < 0.05).

The initial adhesiveness values of yogurt-type fermented soy beverage samples depended on the dairy starter culture used (Figures 5, 6), independent of the level of dry matter concentration in soy beverage samples. The determination of adhesiveness during refrigerated storage revealed a significant influence on texture analysis parameters for both YOMIX-207 and YC-X16 fermented soy beverage samples. It is important to note that the measured adhesiveness values of yogurt-type fermented soy beverage remained relatively constant between 7 and 14 days of refrigerated storage.

The textural properties of yogurt-type fermented soy beverage are important indicators of their quality, as highlighted by Xu et al. (2022). However, Xu et al. (2022) found that fermentation with different probiotic strains had little effect on the texture of yogurt-type fermented soy beverages. Nevertheless, the textural properties of the samples were consistent with WHC. Therefore, in the present study, the focus was on the effect of soy beverage dry matter concentration on textural characteristics, and this effect was tested only on beverages fermented with two selected dairy starter cultures. Existing literature studies have demonstrated that the dry matter content significantly affects the textural characteristics of dairy yogurts (Shaker et al., 2000; Paseephol et al., 2008; Mitra et al., 2022; Gurskiy, 2023). In line with these findings, our research has shown that both hardness and adhesiveness are dependent on the dry matter content of the soy beverage, and these parameters exhibit changes in their values during the refrigerated storage of fermented soy beverage samples. The most significant changes were observed during the first and second weeks of refrigerated storage. Both dairy starter cultures used in these experiments (YOMIX-207 and YC-X16) were claimed to possess high viscosity properties. It should be emphasized that the high viscosity properties declared by the manufacturers refer to the fermentation of milk and not a soy beverage. It is worth also noting that dairy starter cultures capable of creating desirable textural characteristics in fermented products are often capable of producing EPS (Duboc and Mollet, 2001; Li et al., 2014; Xu et al., 2019). In fact, dairy starter cultures that produce EPS have the potential to replace stabilizers, as suggested by Hassan and Awad (2005).

In addition, it is worth noting that we used heat-treated soy beverage (heated to 121 ° C) in our experiments. The application of 121°C to soy can actually affect the functionality of soy proteins. During heated at 121°C, soy proteins can undergo denaturation and conformational changes related to changes in their tertiary and quaternary structures, which can lead to loss of functionality (Kilara and Sharkasi, 2009; Ingrassia et al., 2017). Furthermore, denatured proteins can lose their ability to form stable structures and no longer perform their normal functions in food.

### 3.6. The carbohydrates content changes during refrigerated storage of yogurt-type fermented soy beverage

Monitoring changes in carbohydrate content during storage is a valuable approach to assess product stability, as it directly impacts the sensory properties of the beverages. In this study, we conducted an analysis of carbohydrate content of the base soy beverage and the yogurt-type fermented soy beverages, with a focus on the samples fermented by the two selected dairy starter cultures: YC-X16 and YOMIX-207. In interpreting these results, we examined the relationships between lactic acid bacteria growth dynamics and the carbohydrate content in the samples. The base soy beverage initially contained varying amounts of fructose, galactose, glucose, sucrose, raffinose, stachyose, and verbascose. Specifically, the average carbohydrate content of the base soy beverage was 3.1 mg % ± 0.8 fructose, 15.4 mg % ± 5.1 galactose, 22.9 mg % ± 0.5 glucose, 3.6 mg % ± 0.9 sucrose, 2.8 g % ± 0.0 raffinose, 25.2 mg % ± 1.1 stachyose, and 2.7 mg % ± 0.3 verbascose. Fermentation of the base soy beverage resulted in a change in the content of assayed sugars, and the direction of these changes depended on the type of starter culture used (Table 5). During refrigerated storage of the samples, both the storage time and the type of starter culture used significantly influenced changes in certain carbohydrates’ content in the yogurt-type fermented soy beverage.

**Table 5:** Changes in sugar content [mg %] changes during refrigerated storage of yogurt-type fermented soy beverage.

Sugar	Fermented by YOMIX-207 starter culture						Fermented by YC-X16 starter culture					
Storage time [day]	0	7	14	21	28	35	0	7	14	21	28	35
Fructose	4.8 ± 0.7^a^	6.5 ± 1.4^a^	10.4 ± 1.7^b^	10.3 ± 1.7^b^	10.3 ± 1.6^b^	12.9 ± 2.0^b^	4.7 ± 0.8^a^	4.8 ± 0.7^a^	4.8 ± 0.7^a^	4.9 ± 0.8^a^	5.0 ± 0.7^a^	5.0 ± 0.8^a^
Galactose	19.4 ± 3.9^a^	21.8 ± 4.9^a^	22.9 ± 4.2^a^	20.4 ± 3.4^a^	18.8 ± 2.8^a^	19.8 ± 2.7^a^	20.3 ± 5.1^a^	22.4 ± 5.4^a^	24.9 ± 6.0^a^	27.3 ± 6.9^a^	27.0 ± 7.0^a^	29.8 ± 7.6^a^
Glucose	26.0 ± 0.3^e^	30.4 ± 1.1^f,g^	31.3 ± 1.9^g^	29.5 ± 0.4^g^	26.4 ± 0.5^e,f^	28.7 ± 0.7^f^	21.3 ± 0.7^d^	19.3 ± 0.4^c,d^	16.9 ± 1.0^e^	13.9 ± 0.5^b^	11.6 ± 0.4^b^	9.1 ± 0.4^a^
Sucrose	0.9 ± 0.5^a^	1.0 ± 0.1^a^	0.8 ± 0.5^a^	0.9 ± 0.4^a^	0.8 ± 0.1^a^	0.8 ± 0.1^a^	2.9 ± 0.5^c^	3.1 ± 0.5^c^	2.5 ± 0.5^b,c^	2.6 ± 0.4^b,c^	2.1 ± 0.4^b^	2.0 ± 0.4^b^
Raffinose	3.0 ± 0.6^b^	1.9 ± 0.0^a^	2.1 ± 0.2^a,b^	1.6 ± 0.2^a^	1.8 ± 0.2^a^	1.3 ± 0.1^a^	4.1 ± 0.6^c^	4.1 ± 0.5^c^	4.2 ± 0.6^c^	4.2 ± 0.6^c^	4.3 ± 0.6^c^	4.3 ± 0.6^c^
Stachyose	21.4 ± 0.1^c^	16.6 ± 0.9^b^	13.5 ± 0.1^a,b^	15.4 ± 0.5^b^	12.1 ± 0.0^a,b^	9.9 ± 0.2^a^	20.0 ± 3.2^c^	17.3 ± 2.7^b,c^	16.5 ± 2.6^b^	16.0 ± 3.0^b^	16.0 ± 3.0^b^	14.4 ± 2.9^a,b^
Verbascose	01.6 ± 0.4^b^	0.0 ± 0.0^a^	0.0 ± 0.0^a^	0.0 ± 0.0^a^	0.0 ± 0.0^a^	0.0 ± 0.0^a^	5.5 ± 2.5^c^	0.0 ± 0.0^a^	0.0 ± 0.0^a^	0.0 ± 0.0^a^	0.0 ± 0.0^a^	0.0 ± 0.0^a^

Different letters in the same row indicate significant differences (*p* < 0.05).

Raffinose and stachyose are α-galactosides that are not digested by the human gut (Leblanc et al., 2004). A wide range of LAB species has been shown to have α-galactosidase activity and the ability to break down raffinose and stachyose in soy (Mital and Steinkraus, 1975; Donkor et al., 2007; Hati et al., 2014) as well as in pea and bean flour (Duszkiewicz-Reinhard, Gujska, and Khan Duszkiewicz-Reinhard et al., 1994). Also, bacteria of the genus *Bifidobacterium* species, in particular, exhibit high α-galactosidase activity and are commonly used in biotechnological processes involving soy milk as a substrate. The starter cultures containing not only *L. delbrueckii* species and *S. thermophilus* but also additional microflora such as bifidobacteria are especially beneficial due to the symbiotic relationship between these microorganisms (Hou et al., 2000; Mondragón-Bernal, 2012; Ziarno et al., 2019). Research has indicated that certain strains of *Bifidobacterium* preferentially metabolize galacto-oligosaccharides over sucrose during the fermentation of soy beverages (Bordignon et al., 2004). Contrary to dairy starter cultures for yogurt production, *Bifidobacterium* strains have been shown to effectively reduce raffinose and stachyose levels during growth in soy beverages Scalabrini et al. (1998). The time of the fermentation process also plays a significant role in the metabolic activities of the bacteria involved (Granito et al., 2003). Overall, studying changes in carbohydrate content during the refrigerated storage of yogurt-type fermented soy beverage is essential for assessing fermentation processes, shelf life, nutritional value, and overall quality of the product. These investigations provide valuable information for producers and consumers, enabling them to make informed decisions regarding product formulation, storage conditions, and consumption. Furthermore, the breakdown of α-galactosides by LAB strains contributes to the improved nutritional value of fermented plant-based products, as these oligosaccharides naturally occur in such foods (Wang et al., 2003; Shimelis and Rakshit, 2008; Ziarno et al., 2019).

### 3.7. The fatty acid profile during refrigerated storage of yogurt-type fermented soy beverage

This research focuses on investigating the impact of storage on the fatty acid composition of yogurt-type fermented soy beverage, which is an important aspect of their nutritional quality. The study examines changes in the levels of different types of fatty acids, including saturated, monounsaturated, and polyunsaturated fatty acids, during refrigerated storage and evaluates their effect on the nutritional value of the beverage. The findings of this research are valuable for improving the quality of yogurt-type fermented soy beverage and provide useful information for both food manufacturers and consumers. Studies of the fatty acid profile were conducted only on samples of soy beverages fermented by the two specific dairy starter cultures: YC-X16 and YOMIX-207. Caprylic acid (C8:0), capric acid (C10:0), and lauric acid (C12:0) were not detected. However, myristic acid (C14:0), palmitic acid (C16:0), stearic acid (C18:0), eicosanoic acid (C20:0), heneicosanoic acid (C20:1), docosanoic acid (C22:0), tetracosanoic acid (C24:0), oleic acid (C18:1 omega-9), erucic acid (C22:1 omega-9), linoleic acid (C18:2 omega-6), and α-linolenic acid (C18:3 omega-3) were present in variable proportions in the fatty acid profile of fresh samples of yogurt-type fermented soy beverage. The levels of these fatty acids were found to have a statistically significant relationship with the type of dairy starter culture used. The fatty acid profile of yogurt-type fermented soy beverage was influenced by both the starter culture used and the duration of refrigerated storage (Table 6). Statistically significant changes were observed in the proportions of myristic acid (C14:0), palmitic acid (C16:0), and linoleic acid (C18:2 omega-6) in the overall fatty acid composition of the beverages. However, the AI and TI coefficients, which are indicators of the cardiovascular risk associated with dietary fat intake, did not show statistically significant changes. It is worth noting that initially, the AI values differed depending on the starter culture used, with the YOMIX-207 culture showing a mean AI value of 0.13 ± 0.01 and the YC-X16 culture exhibiting a mean AI value of 0.15 ± 0.01. However, during refrigerated storage, these values became statistically nonsignificantly equalized.

**Table 6:** Changes in fatty acid profile [as % of the total fatty acid pool] during refrigerated storage of yogurt-type fermented soy beverage.

Fatty acids [% of the total fatty acid pool]	Fermented by YOMIX-207 starter culture						Fermented by YC-X16 starter culture					
Storage time [day]	0	7	14	21	28	35	0	7	14	21	28	35
Caprylic C8:0	nd	nd	nd	nd	nd	nd	nd	nd	nd	nd	nd	nd
Capric (decanoate) C10:0	nd	nd	nd	nd	nd	nd	nd	nd	nd	nd	nd	nd
Lauric C12:0	nd	nd	nd	nd	nd	nd	nd	nd	nd	nd	nd	nd
Myristic C14:0	0.02 ± 0.03^a^	0.06 ± 0.00^b^	0.07 ± 0.01^b^	0.06 ± 0.03^b^	0.07 ± 0.02^b^	0.08 ± 0.03^b^	0.07 ± 0.00^b^	0.07 ± 0.01^b^	0.05 ± 0.03^a,b^	0.01 ± 0.03^a^	0.03 ± 0.03^a^	0.00 ± 0.01^a^
Palmitic C16:0	10.50 ± 0.54^a^	10.62 ± 0.21^a^	10.95 ± 0.25^a,b^	11.15 ± 0.37^a,b^	11.12 ± 0.49^a,b^	11.08 ± 0.54^a,b^	12.25 ± 1.00^c^	11.80 ± 0.46^b,c^	11.68 ± 0.23^b,c^	11.71 ± 0.56^b,c^	11.96 ± 0.45^b,c^	11.59 ± 0.44^b,c^
Stearic C18:0	4.40 ± 0.50^a^	4.32 ± 0.36^a^	4.62 ± 0.37^a^	4.68 ± 0.38^a^	4.62 ± 0.35^a^	4.48 ± 0.12^a^	4.03 ± 0.48^a^	4.41 ± 0.48^a^	4.44 ± 0.36^a^	4.42 ± 0.43^a^	4.70 ± 0.18^a^	4.74 ± 0.15^a^
Oleic C18:1 omega-9	22.68 ± 0.48^a^	22.93 ± 0.12^a^	22.58 ± 0.32^a^	22.62 ± 0.63^a^	22.57 ± 0.56^a^	22.44 ± 0.47^a^	22.61 ± 0.59^a^	22.26 ± 0.99^a^	22.33 ± 0.55^a^	22.19 ± 1.33^a^	21.65 ± 0.63^a^	22.71 ± 0.74^a^
Linoleic C18:2 omega-6	53.91 ± 0.87^b^	53.39 ± 0.29^a,b^	53.46 ± 0.42^a,b^	53.29 ± 0.56^a,b^	53.01 ± 0.83^a,b^	52.99 ± 0.60^a,b^	52.47 ± 1.07^a^	52.99 ± 0.59^a,b^	53.11 ± 0.34^a,b^	52.93 ± 0.49^a,b^	52.67 ± 0.50^a,b^	52.26 ± 0.31^a,b^
α-linolenic C18:3 omega-3	6.91 ± 0.41^a^	6.86 ± 0.50^a^	6.70 ± 0.38^a^	6.70 ± 0.37^a^	6.91 ± 0.38^a^	7.18 ± 0.41^a^	6.90 ± 0.46^a^	6.71 ± 0.39^a^	6.73 ± 0.40^a^	6.71 ± 0.40^a^	6.64 ± 0.33^a^	6.93 ± 0.55^a^
Eicosanoic C20:0	0.54 ± 0.06^a^	0.58 ± 0.07^a^	0.58 ± 0.09^a^	0.55 ± 0.07^a^	0.57 ± 0.09^a^	0.57 ± 0.12^a^	0.49 ± 0.02^a^	0.51 ± 0.02^a^	0.53 ± 0.06^a^	0.57 ± 0.04^a^	0.62 ± 0.10^a^	0.54 ± 0.04^a^
Heneicosanoic C20:1	0.44 ± 0.09^a^	0.49 ± 0.07^a^	0.41 ± 0.03^a^	0.45 ± 0.20^a^	0.43 ± 0.07^a^	0.45 ± 0.05^a^	0.43 ± 0.02^a^	0.43 ± 0.07^a^	0.41 ± 0.03^a^	0.50 ± 0.19^a^	0.47 ± 0.11^a^	0.37 ± 0.10^a^
Docosanoic C22:0	0.21 ± 0.05^a^	0.25 ± 0.07^a^	0.20 ± 0.02^a^	0.18 ± 0.03^a^	0.23 ± 0.03^a^	0.20 ± 0.06^a^	0.21 ± 0.04^a^	0.19 ± 0.05^a^	0.19 ± 0.03^a^	0.27 ± 0.14^a^	0.31 ± 0.13^a^	0.21 ± 0.04^a^
Erucic C22:1 omega-9	0.28 ± 0.05^a^	0.30 ± 0.09^a^	0.28 ± 0.09^a^	0.28 ± 0.08^a^	0.30 ± 0.10^a^	0.33 ± 0.12^a^	0.22 ± 0.04^a^	0.25 ± 0.01^a^	0.24 ± 0.05^a^	0.26 ± 0.06^a^	0.30 ± 0.08^a^	0.24 ± 0.04^a^
Tetracosanoic C24:0	0.09 ± 0.02^a^	0.06 ± 0.05^a^	0.06 ± 0.04^a^	0.02 ± 0.04^a^	0.06 ± 0.03^a^	0.02 ± 0.01^a^	0.02 ± 0.05^a^	0.07 ± 0.02^a^	0.03 ± 0.05^a^	0.03 ± 0.04^a^	0.05 ± 0.04^a^	0.03 ± 0.04^a^
AI	0.13 ± 0.01^a^	0.13 ± 0.00^a^	0.13 ± 0.00^a^	0.14 ± 0.01^a,b^	0.14 ± 0.01^a,b^	0.14 ± 0.01^a,b^	0.15 ± 0.01^b^	0.15 ± 0.01^b^	0.14 ± 0.00^a,b^	0.14 ± 0.01^a,b^	0.15 ± 0.01^a,b^	0.14 ± 0.01^a,b^
TI	0.25 ± 0.02^a^	0.25 ± 0.02^a^	0.27 ± 0.02^a^	0.27 ± 0.01^a^	0.27 ± 0.01^a^	0.26 ± 0.01^a^	0.28 ± 0.01^a^	0.28 ± 0.02^a^	0.28 ± 0.01^a^	0.28 ± 0.01^a^	0.29 ± 0.01^a^	0.28 ± 0.01^a^

AI, atherogenic index; TI, thrombogenic index; nd, not detected. Different letters in the same row indicate significant differences (*p* < 0.05).

This is consistent with Zaręba’s study (Zaręba, 2009) on the fatty acid profile of soy beverages fermented by different lactic acid bacteria (various strains of yogurt bacteria from the species *L. delbrueckii* subsp. *bulgaricus* and *S. thermophilus*). The cited research showed that the type of yogurt starter culture had no statistically significant effect on the fatty acid profile during fermentation. However, changes in the fatty acid profile were observed only during refrigerated storage of yogurt-type fermented soy beverage. The analysis of fatty acid composition confirmed the presence of significant amounts of acids with chain lengths ranging from C16:0 to C22:0. Zaręba (2009) identified several fatty acids in the study, including lauryl, palmitic, palmitoleic, margaric, nonadecanoic, oleic, vaccenic, linoleic, arachidic, and α-linolenic acids. Among the major fatty acids in yogurt-type fermented soy beverages studied by Zaręba (2009) were linoleic, oleic, palmitic, and α-linolenic acids. The cited literature data reported a reduction in linoleic and oleic fatty acids in refrigerated storage of fermented soy beverage samples. The cited author explains the observed changes in the enzymatic activity of lactic acid bacteria (for example, some lactobacilli can use linoleic acid as substrate for CLA synthesis) and its variability in an environment with a pH that changes over time. This explanation is supported by the literature data from (Collins et al., 2003a,b; Donkor et al., 2007; Liavonchanka and Feussner, 2008; Ares-Yebra et al., 2019; Aziz et al., 2022; Abedin et al., 2023). The same may also apply to the present study, as significant changes in the fatty acid profile were observed during the first few weeks of refrigerated storage of fermented soy beverage samples when the pH values of the products were still changing, and lactic acid bacteria were characterized by high levels of viability.

The AI and TI are interesting parameters that are calculated through the fatty acid profile. Both indicators have value in predicting the health value of food (Florence et al., 2012; Balthazar et al., 2016; Berg and McCarthy, 2022). Regarding dairy products, which are often associated with higher fat content and potential atherogenic effects, Balthazar et al. (2016) conducted a study on sheep’s milk yogurt and reported a nonsignificant decrease in the AI during storage. The AI values decreased from 3.30 on the first day to 2.93 after 28 days, while the TI values decreased from 4.38 to 3.45 over the same period. In contrast, Ardabilchi Marand et al. (2020) explored the impact of flaxseed powder addition on AI and TI values in fermented beverages. Their findings demonstrated a significant decrease in both AI (from 2.04 to 0.95) and TI (from 2.4 to 0.41) values upon the addition of flaxseed powder. This reduction indicated an improvement in the health outcomes of the fermented beverage. In the present study, the obtained AI and TI values are comparable to those reported by Ardabilchi Marand et al. (2020) investigated the effects of adding flaxseed powder on nutritional aspects (including associated health lipid indices such as atherogenic and thrombogenic indices) of fortified yogurt samples during 21 days of cold storage. It is worth noting that the cited researchers used another dairy starter culture in the research, namely YC-X11, which contained *S. thermophilus* and *L. delbrueckii* subsp. *bulgaricus*.

## 4. Conclusion and future research direction

The traditional production of yogurt-type fermented soy beverages relies on natural bacterial cultures present in soybeans. However, this process lacks control and often leads to inconsistent product quality, making it challenging for commercialization purposes. To address this issue, the use of dairy starter cultures has been introduced, allowing for greater control over the fermentation process and ensuring a more consistent and uniform product of established quality. The production of yogurt-type fermented soy beverages using dairy starter cultures offers several advantages, including the ability to control the fermentation process and produce a high-quality product that can be easily scaled up and commercialized.

Fermentation is a key step in the production of yogurt-type fermented soy beverage, with selected dairy starter cultures being particularly important. For dairy starter cultures consisted of typical yogurt microorganisms and with or without additional microflora the maximum acidification rate ranged from 0.006 to 0.013 depending on the starter culture tested. This research focuses on understanding dairy starter culture choice and fermentation time as key parameters affecting the fermentation process of soy beverages. By understanding the interplay of both key parameters, we can achieve the desired end product quality characteristics and textures, of the final product and proper shelf life during storage time as well as proper shelf life during storage time. In addition to taste and texture, the nutritional value of soya yogurt-type beverages is also of great interest to consumers. In this research, we demonstrated the influence of dairy starter culture choice on the sensory and nutritional profiles of fermented soy beverages. The choice of culture has a significant impact on various aspects, including acidification kinetics, live cell populations, pH values, water-holding capacity, texture properties, and carbohydrate content of the beverages. These results provide valuable insights for optimizing the production process and improving the quality of yogurt-type fermented soy beverage. In conclusion, the results of this study highlight the importance of selecting appropriate dairy starter cultures for the production of yogurt-type fermented soy beverage.

Although the potential of using dairy starter cultures in soy beverage fermentation holds promise, several challenges and limitations need attention. Key factors include culture adaptation, stability during fermentation, as well as addressing potential hypoallergenicity concerns and the presence of sugars from the raffinose family oligosaccharides (RFO) that cause gut discomfort for many consumers. It is crucial to consider emerging trends and develop new dairy starter cultures specifically designed for fermenting plant-based raw materials, given the rising demand for plant-based alternatives and consumer preferences. By comprehending the science behind this approach and addressing the associated challenges, we can pave the way for developing high-quality, nutritious, and appealing plant-based products that cater to the demands of health-conscious consumers. This finding holds the potential to pave the way for the creation of plant-based dairy alternatives that are not only tastier but also more nutritious.

## Data availability statement

The original contributions presented in the study are included in the article/supplementary material, further inquiries can be directed to the corresponding author.

## Author contributions

MZ, DZ, IŚ, and MK: data curation, conceptualization, formal analysis, visualization, writing – review and editing, and supervision. MZ: project administration, funding acquisition, and supervision. All authors contributed to the article and approved the submitted version.

## Conflict of interest

The authors declare that the research was conducted in the absence of any commercial or financial relationships that could be construed as a potential conflict of interest.

## Publisher’s note

All claims expressed in this article are solely those of the authors and do not necessarily represent those of their affiliated organizations, or those of the publisher, the editors and the reviewers. Any product that may be evaluated in this article, or claim that may be made by its manufacturer, is not guaranteed or endorsed by the publisher.
